# Working from home during the COVID 19 pandemic: a longitudinal examination of employees’ sense of community and social support and impacts on self-rated health

**DOI:** 10.1186/s12889-022-14904-0

**Published:** 2023-01-03

**Authors:** Melissa Graham, Katrina A. Lambert, Victoria Weale, Rwth Stuckey, Jodi Oakman

**Affiliations:** Department of Public Health, School of Psychology and Public Health, La Trobe University, Bundoora, VIC 3086 Australia

**Keywords:** COVID 19, Work from home, Sense of community, Social support, Workplace, Self-rated general health

## Abstract

**Background:**

The COVID 19 pandemic resulted in the introduction of public health measures including mandated and recommended work from home orders to reduce transmission. This provided a unique opportunity to examine sense of community and social support within the workplace and self-rated general health. This paper examines employees’ workplace sense of community and social support across one year of the COVID 19 pandemic and associated self-rated general health.

**Methods:**

Analysis of longitudinal data (October 2020, May 2021, and November 2021) from the Employees Working from Home study conducted in Victoria, Australia during the COVID 19 pandemic was undertaken. Trajectory analyses were used to describe workplace sense of community and social support over time. Multinomial logistic regression was used to determine the associations between demographics, gender, caring responsibilities, and group membership based on the Growth Mixture Modelling. Generalised Mixed Models were used to measure effects of sense of community and social support on self-rated health.

**Results:**

Increasing sense of community and social support in the workplace resulted in increased self-rated health. Trajectory analysis found two stable and distinct groups for sense of community. Social support varied with time; however, trajectory membership was not dependent on gender or caring responsibilities and had no relationship with return to the office.

**Conclusion:**

Sense of community and social support in the workplace are important determinants of employees’ health, and as such, workplace strategies to improve sense of community and social support are required not only for employees working from home, but also those who have returned to the office, particularly as hybrid work arrangements become more common.

**Supplementary Information:**

The online version contains supplementary material available at 10.1186/s12889-022-14904-0.

## Introduction

The declaration of the COVID 19 pandemic by the World Health Organization in March 2020 [1] led to the implementation of public health measures by governments which fundamentally changed the way office work was undertaken. Prior to the COVID 19 pandemic, work from home (WFH), often called remote work, telework, or telecommuting, was largely viewed as a negotiated arrangement to support work-life integration [2, 3]. In response to the COVID 19 pandemic, governments mandated WFH for all employees who were able to undertake their role from home. In Victoria, Australia WFH mandates were in place from March 2020 until early November 2021, with the exception of three brief periods of recommended WFH in May 2020, December 2020 and January 2021, and December 2021 when the mandate was replaced with a recommendation to WFH if able to do so [4].

The rapid change to WFH challenged the previously held belief that employees need to be co-located in the office to establish and maintain connections, networks, provide and receive support, and to feel a sense of community. Prior research has argued the potential loss of social connections, and therefore support and sense of community, due to remote working [5, 6]. However, mandated WFH orders disrupted the usual means of establishing sense of community and social support within the workplace, with potential impacts on general health. Arguably, a strong sense of community and high levels of social support in the workplace are key to successfully managing employees working from home. This paper addresses a gap in the existing evidence base by examining sense of community and social support across one year of the pandemic and mandated WFH, and if sense of community and social support in the workplace are associated with self-rated general health. Sense of community and social support and impacts on self-rated general health while working from home has not previously been addressed in a COVID 19 context with public health restrictions in place. Understanding the implications of working from home on workplace sense of community, social support and associated health has becoming increasingly more important as workplaces shift to hybrid ways of working. This paper offers insights into hybrid working which is increasing being adopted as standard workplace practice.

### Sense of community and social support in the workplace

Sense of community in the workplace has been described in several ways; however, the main components relate to relationships with colleagues and managers, communication, and networking to create a sense of belonging, mattering, connection, and support, and having needs meet through resources and supportive organisational policies [7–11]. High involvement management, that can be a source of support, may also contribute to positive outcomes, such as improved self-rated general health [12]. For the purpose of this study, we adopt the definition of sense of community at work from the Copenhagen Psychosocial Questionnaire (COPSOQ) [13], that is, “feeling of being part of the group of employees at the workplace, e.g., if employees relations are good and if they work well together”.

Sense of community has been found to be associated with, and a predictor of, health and wellbeing within the workplace. However, much of the research both prior to and during the COVID 19 pandemic has focused on wellbeing, quality of life, anxiety, depression, stress, and burnout, with little attention paid to general health. A US study conducted with 369 healthcare employees found sense of community could predict psychological wellbeing [14, 15]. Similarly, a cross-sectional study with a sample of 873 healthcare workers in China found poor or no sense of community was associated with poor quality life and psychological health [16]. Sense of belonging and mattering have been found to be lower among teleworkers compared to office-based workers [17] leading to social isolation which can impact one’s health and wellbeing. A cross-sectional study conducted in the US with a sample of 5,550 participants found high rates of stress, anxiety, depression, work exhaustion, burnout, and poor wellbeing among those working from home during the COVID 19 pandemic, and health and wellbeing outcomes were associated with perceptions of family supportive supervisors. Organisational policies and supportive supervisor behaviour (such as making employees feel comfortable to discuss and manage work-life conflict) can improve employee wellbeing [18].

Closely related to sense of community is workplace social support. Social support in the workplace is essential to forming and maintaining collegial supportive relationships both among employees and between employees and employers. Social support in the workplace refers to the availability of tangible instrumental and informational supports as well as non-tangible emotional and appraisal supports [19] and can occur at the organisational level, and among and between supervisors / employers, and colleagues, whereby the possibility of obtaining support, if required, is available [13]. Social support can buffer the relationship between negative or health detrimental experiences (for example, stress) and health and wellbeing outcomes, which is critical in the context of WFH mandates during COVID 19 [20].

Social support in the workplace has been associated with stress, musculoskeletal pain, physical and mental health, quality of life, and wellbeing [16, 21–26]. Research examining COVID 19 related anxiety among nurses and university staff suggests higher levels of social and organisational support are associated with less COVID 19 anxiety [27, 28]. Similarly, a longitudinal study in Finland found high social support was associated with lower COVID 19 anxiety [29]. Wang and colleagues’ [30] sequential mixed methods study with a sample of Chinese employees working from home suggests workplace social support is an important resource to address the challenges of remote working including mitigating loneliness and improving wellbeing. Further, they argue social support has become more important during the COVID 19 pandemic as it has positive impacts on wellbeing through its buffering effect [30].

To the authors’ knowledge no research has examined sense of community or social support in the workplace and self-rated general health during mandated WFH, yet self-rated general health provides important insight into, and is a predictor, of health. Self-rated general health measures subjective health status which incorporates physical, mental, social, biological, and functional aspects. It is a useful measure as it is considered an independent predictor of mortality and appears to do so universally across populations. Self-rated general health is an independent non-casual predictor of mortality as the self-assessor is able to consider the meaning of the concept health to them and the objective information available to the self-assessor about their health [31–34]. Sense of community and social support, known determinants of health [35, 36], within the workplace are important as they have the potential to mitigate negative health consequences; however, limited research has examined these factors in the workplace context and to the authors’ knowledge, none which has considered mandated WFH. COVID 19 has provided a unique opportunity to examine workplace sense of community and social support over time during mandated WFH, as opposed to voluntary WFH, and subsequent self-rated general health. Further, given the shift to more flexible hybrid ways of working, including increased working from home, understanding the role of workplace social support and sense of community and their relationship with self-rated general health is important for both employers and employees to inform policy development, support mechanisms, and provide insight to assist employers with how best to support their employees.

First, a trajectory analysis of sense of community and social support was conducted to answer the following research questions: did employees’ sense of community and social support change across one year of full or partial WFH during the COVID 19 pandemic; and are there groups with distinct trajectories? Second, sense of community, social support, and self-rated general health were examined to answer following research questions: are workplace sense of community and social support associated with self-rated general health over one year of the COVID 19 pandemic; and are any associations modified by gender, caring responsibilities, and changing number of days working at home? It was hypothesised: sense of community and social support trajectories would follow a cubic pattern in line with public health restrictions; and higher levels of sense of community and social support are associated with better self-rated general health.

## Methods

This study used data collected from the Employees Working from Home (EWFH) study conducted in Australia during the COVID 19 pandemic from October 2020 to November 2021. Sampling and recruitment for the EWFH study have been described elsewhere [37]. Briefly, convenience sampling was used to recruit a sample of Australian adults aged 18 or more years who worked from home two or more days per week during the COVID 19 pandemic. Recruitment occurred via Facebook’s paid service, professional and personal networks, the La Trobe University Facebook page, and LinkedIn. Data were collected by questionnaire at three time points with all participants who consented at baseline to be recontacted invited to complete Waves 2 and 3. Responses rates at Waves 2 and 3 were 67% and 53% respectively. Methods were conducted in accordance with the Australian Code for the Responsible Conduct of Research (2018). All participants provided informed consent to participate. Ethics approval was obtained through La Trobe University Human Ethics Research Committee (HEC20388).

The current analysis focuses on data from Victoria, Australia (84%; *n* = 658 of total sample) with Wave 1 (baseline) data sourced during the height of Victoria’s second wave of COVID 19 in October 2020. Restrictions in place at the time required those who could work at home to do so, while childcare and schools were available only to children of essential workers. Return to the office at the time of Waves 2 and 3 was variable. As such, return-to-work trajectories were calculated using Growth Mixture Modelling (GMM) analyses to identify latent classes with different growth trajectories of the ratio of days worked at a participants’ usual place of work outside the home over total number of days worked across the three timepoints (*n* = 399). Three distinct classes were identified (Figure S1), those who worked from home at all timepoints (35.8% *n* = 143), those who had partially returned to the office at Wave 2 (May 2021) but were working from home at Wave 3 (October 2021; 59.4% *n *= 237), and those who had fully returned to their usual place of work (4.8% *n* = 19).

Sense of community and social support were measured using items from the COPSOQ [13]. Sense of community was measured by two items with items rated on a five-point scale from never/hardly ever (1) to always (5). An example item is “Is there a good atmosphere between you and your colleagues?“ Self-rated general health was measured by response to the item “In general, would you say your health is:”, with participants selecting an option from poor (1) to excellent (5).

Potential effect modification by gender and caring responsibilities were considered. Gender was based on the item “Are you: Male, Female, Other.” Four participants who reported their gender as “Other” were excluded from the gender analysis. Participants were considered to have caring responsibilities if they answered, “With one or more adults AND children aged 0–18 years” or “With one or more children aged 0–18 years (no other adults)” to the question “Which of the following best describes your usual living arrangements?” or answered “Yes, adult(s) living with me”, “Yes, adult(s) living elsewhere”, or “Other” to the question “Do you have caring responsibilities other than children?” An interactive effect of gender and caring responsibilities was also considered.

Models were adjusted for age, work hours, occupation classification, and home workspace. Age was based on the item “What is your age group?” 18–25 years; 26–35 years; 36–45 years; 46–55 years; 56 years and over. The categories were then collapsed to 18–35 years; 36–45 years; 46–55 years; 56 years and over. Work hours was based on the item “What are your usual working hours (average per week)?” with answers above 35 h per week considered full-time. Occupation classification was used as a proxy for income and education and based on the item “Which best describes your current role?” from the Australian and New Zealand Standard Classification of Occupations [38] and categorised as professional, managers and other. Workspace was based on the item “When you are working at home, where do you usually work?”. Three response options were provided and coded as follows: Wherever — “I just find a place somewhere that’s free, such as on the kitchen table or other place”; Separate — “I have my own place in a separate room by myself”; and Interruptions — “I have my own place but in a room that can be busy with other people.”.

### Data analysis

To describe the course of sense of community and social support over the study period GMM analyses were used to identify latent classes with different growth trajectories over the three time points. These models are less restrictive than a latent class analysis, as the GMM accounts for between-subject heterogeneity by including random effects. Participants were required to have completed at least two timepoints to be included in the trajectory modelling. GMM models with one to five classes were examined, with each model being run 50 times with different starting values to ensure the optimal solution was found instead of local maxima. The optimal solutions for each class number were compared and the Bayesian information criterion used to select the best fit model [39]. Participants were matched to a latent class using posterior probabilities, with individuals allocated to the group for which the probability was the highest. Trajectory analyses were run with the ‘hlme’ function from the R package ‘lcmm’ [40]. Where three or more groups were identified, multinomial logistic regression was used to determine the associations between demographics, gender, caring responsibilities, and group membership based on the GMM. Odds ratios (OR) with 95% confidence intervals (CI) were calculated, comparing membership in each group to the highest stable group.

Effects of sense of community and social support on self-rated general health were calculated with cumulative link mixed models treating general health as an ordinal factor variable and random slope ID using the R package “ordinal” [41]. This analysis used all observations and did not require a second time point. Correlations between multiple observations from the same individual are accounted for with the random slope in the mixed model. Potential effect modification by gender and caring responsibilities was explored by interaction and stratified analysis.

## Results

**Fig. 1:**
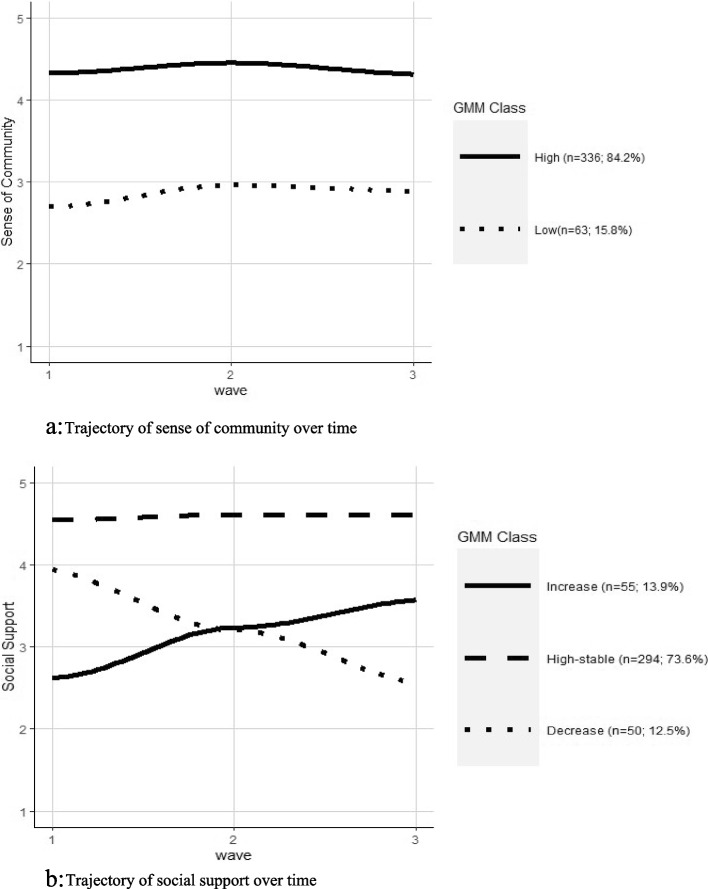
** a** Trajectory of sense of community over time.** b** Trajectory of social support over time

A two-class solution was selected as the best fit for the trajectory of sense of community, while a three-class solution was optimal for the trajectories of social support (Table S1). Sense of community contained two stable groups – high (84.2%) and low (15.8%: Fig. 1a). Both groups show a slight increase at Wave 2 but are generally time invariant. Social support varied with the largest group being high stable (73.6%) and remained constant over time (Fig. 1b). Two other groups were identified – increase (13.8%) and decrease (12.5%).

There were no statistically significant differences in gender or caring responsibilities between the identified social support trajectories (Table 1). Similarly, there was no relationship between return to the office and social support trajectories (χ^2^ = 4.9268, *p*-value = 0.2949).

**Table 1:** Trajectories of social support and multinomial logistic regression associations between age, gender, and caring responsibilities

	**High-Stable (*n* = 294)**	**Increase (*n* = 55)**	**Decrease (*n* = 50)**	**Increase vs High** OR (95%CI)	**Decrease vs High** OR (95%CI)
**Age**					
18–35	73 (24.83%)	17 (31.48%)	17 (34.00%)	Ref	Ref
36–55	178 (60.54%)	30 (55.56%)	25 (50.00%)	0.71 (0.38, 1.33)	0.62 (0.32, 1.18)
56 +	43 (14.63%)	7 (12.96%)	8 (16.00%)	0.72 (0.29, 1.78)	0.86 (0.36, 2.07)
**Gender**					
Male	60 (20.41%)	14 (25.45%)	15 (30.00%)	Ref	Ref
Female	233 (79.25%)	40 (72.73%)	33 (66.00%)	0.76 (0.40, 1.44)	0.58 (0.30, 1.11)
Other	1 (0.34%)	1 (1.82%)	2 (4.00%)		
**Domestic Arrangements**					
Single person household	36 (12.24%)	9 (16.36%)	6 (12.00%)	Ref	Ref
Adults only	127 (43.20%)	24 (43.64%)	21 (42.00%)	0.74 (0.33, 1.67)	0.89 (0.36, 2.25)
Dependents	131 (44.56%)	22 (40.00%)	23 (46.00%)	0.66 (0.29, 1.49)	0.98 (0.39, 2.44)
**Number of Children**					
None	186 (63.27%)	38 (69.09%)	30 (60.00%)	Ref	Ref
1	33 (11.22%)	5 (9.09%)	7 (14.00%)	0.68 (0.25, 1.84)	1.4 (0.60, 3.27)
2	60 (20.41%)	11 (20.00%)	9 (18.00%)	0.91 (0.45, 1.83)	0.97 (0.45, 2.07)
3 or more	15 (5.10%)	1 (1.82%)	4 (8.00%)	0.61 (0.14, 2.77)	1.57 (0.49, 5.00)
**Interaction sex and caring**					
Male w/ care	24 (8.19%)	3 (5.45%)	8 (16.00%)	Ref	Ref
Male w/o care	35 (11.95%)	11 (20.00%)	8 (16.00%)	2.84 (0.73, 11.05)	0.71 (0.24, 2.13)
Female w/o care	160 (54.61%)	29 (52.73%)	21 (42.00%)	1.62 (0.46, 5.68)	0.45 (0.18, 1.11)
Female w/ care	74 (25.26%)	12 (21.82%)	13 (26.00%)	1.48 (0.39, 5.60)	0.56 (0.21, 1.48)

Increasing sense of community (OR:1.88 95% CI 1.48, 2.38) and social support (OR: 1.65 95% CI:1.31, 2.08) increased the odds of rating self-rated health as excellent after adjustment for age, work hours, occupation classification, and home workspace. These relationships were not modified by gender or caring responsibility (Table 2) nor the interaction between gender and caring responsibility (Table 3).

**Table 2:** Potential effect modification by gender or caring responsibility on relationships with self-rated general health^a^

	Male	Female	No Caring Responsibilities	Caring Responsibilities
Sense of community OR (95%CI)	1.745 (1.08, 2.82)	1.878 (1.42, 2.48)	1.800 (1.37, 2.36)	2.278 (1.54, 3.38)
*p*-value (interaction)	Ref	0.806	Ref	0.299
*p*-value (effect)	0.023	< 0.001	< 0.001	< 0.001
Social support OR (95%CI)	1.615 (0.97, 2.69)	1.675 (1.29, 2.19)	1.636 (1.22, 2.20)	1.704 (1.20, 2.43)
*p*-value (interaction)	Ref	0.850	Ref	0.914
*p*-value (effect)	0.065	< 0.001	0.001	0.003

^a^All models adjusted for age, work hours, occupation classification, and home workspace

**Table 3:** Potential effect modification by combined gender and caring responsibility on relationships with self-rated general health^a^

	Male with caring responsibilities	Male without caring responsibilities	Female without caring responsibilities	Female with caring responsibilities
Sense of community OR (95%CI)	1.630 (0.69, 3.84)	1.923 (1.07, 3.47)	1.731 (1.22, 2.46)	1.930 (1.27, 2.94)
*p*-value (interaction)	Ref	0.592	0.899	0.226
*p*-value (effect)	0.263	0.029	0.002	0.002
Social support OR (95%CI)	1.462 (0.63, 3.41)	1.835 (0.91, 3.72)	1.711 (1.21, 2.42)	1.721 (1.16, 2.56)
*p*-value (interaction)	Ref	0.490	0.666	0.498
*p*-value (effect)	0.377	0.092	0.002	0.007

^a^All models adjusted for age, work hours, occupation classification, and home workspace

## Discussion

This aims of this study were twofold. First, we examined employees’ sense of community and social support over one year of the COVID 19 pandemic when WFH was predominantly mandated for most office workers in Victoria, Australia. Prior research argues WFH is detrimental to both workplace sense of community and social support. Given this, it was posited that as WFH mandates tempered with a decline in new COVID 19 cases and some office workers had at least a partial return to the office (for example, hybrid work arrangements whereby their time was split between the office and working from home; Wave 2 data), there would be an improvement in sense of community and social support. However, sense of community remained stable with two distinct groups, high and low sense of community. Sense of community, in this study, remained unchanged regardless of whether participants were working from home, hybrid working, or had a full return to the office, and therefore was not influenced by the location of work. A possible explanation is that employees’ sense of community was well established prior to the COVID 19 pandemic and consequently maintained during working from home. While sense of community did improve slightly at Wave 2, this was not statistically significant. A study conducted in the Netherlands with a large multinational organisation found employees felt isolated from their work community initially during mandated WFH; however, this decreased over time (from March through to May 2020) ]^42^]. In contrast to our findings, research examining Flemish employee perceptions of WFH during COVID 19 found a poorer sense of community among those working from home with participants reporting weaker bonds with their colleagues and feeling less connected to their employer [43]. In our study, it is possible that sense of community remained largely unchanged because of the strategies adopted by organisations to facilitate connection with their employees. Australian qualitative research conducted in the context of the COVID 19 pandemic suggests the adoption of strategies to maintain or improve workplace sense of community included the use of online platforms to improve communication, bonding, networking, and collaboration between staff while working from home [44].

In contrast to sense of community, social support varied. Almost three-quarters of participants reported high levels of social support which was stable over time. However, for 12.5% of participants social support decreased over time while it increased for 13.9% of participants. The changes in social support were not related to demographic characteristics or a return to the office, suggesting the differences were due to workplace characteristics. For example, some workplaces may not have implemented social support strategies with the rapid shift to working from home or did so too late [44]. It is also possible the decline in social support over time is a result of initial efforts made by employers and employees to provide a supportive work environment in unprecedented times which was not sustained due to the protracted period of WFH with supports becoming more strained and shifted towards less supportive actions, for example, increased monitoring and surveillance, micromanaging, and diminished trust [44]. Social support in the workplace has predominantly taken place in the office and it is possible organisations were unable to maintain this due to WFH mandates and no or inadequate alternatives were implemented. For example, many employees worked more flexible hours to balance caring responsibilities, such as childcare and home schooling, and were not necessarily working the same hours as their colleagues and managers. Consequently, the social supports immediately available when in the office were not as accessible whilst working from home. Additionally, seeking and receiving social supports when working from home may require more formally arranged meetings. Opportunities to informally engage with others, comparable to walking around the office to see who might be available, were no longer available. Thus, efforts to maintain support may have been challenging as the use of Zoom became increasingly more fatiguing [44] and workers may have felt they were interrupting if they contacted a colleague or supervisor spontaneously. Conversely, the observed increase in social support over time may reflect employers and employees improving the way they provide support over the course of the WFH mandates.

Similar to the findings from the current study, the limited available evidence on social support in the workplace is mixed, with both positive and negative outcomes reported for employees who WFH. Research conducted in the context of the COVID 19 pandemic suggests working from home creates difficulties in maintaining contact and informal relationships with colleagues [45], and is an obstacle to receiving feedback from employers [3] resulting in increasing workplace isolation and reduced support. However, Moens and colleagues [43] found more than half of employees in their study reported they were well supported by their employers during the rapid shift to WFH. Importantly, supervisor emotional and instrumental support, such as flexibility, has been found to be important for teleworkers both before and during the COVID 19 pandemic [44, 46]. Collins and colleagues’ [46] study conducted prior to the pandemic found social support for teleworkers reduced over time compared to office-based workers. Further, teleworkers sought social support from their existing contacts, that is those they had established prior to teleworking. Similarly, the office-based workers did not seek out relationships with or support from teleworkers. However, in the current study employees had mainly worked together in the office prior to WFH mandates and as such may have felt more able to seek support from their colleagues with whom they had existing relationships, mitigating the decline in their social support. With hybrid models of work now increasing, organisations need to consider how they support those who WFH, in the office, and those who adopt a hybrid model of working.

Second, we examined if sense of community and social support in the workplace were associated with self-rated general health during mandated WFH. We found that both increasing sense of community and social support were associated with higher self-rated general health, and these relationships were retained after adjusting for age, work hours, occupation classification, and home workspace, and were not modified by gender or caring responsibilities. This suggests that organisations should aim to improve sense of community and social support within the workplace, regardless of the workforce characteristics or whether employees are working from home, in the office or a hybrid approach, with potential benefits for employees’ self-rated health.

Organisations need to implement strategies to improve low levels of sense of community and decreasing levels of social support to improve self-rated general health. Strategies may include: supportive organisational policies which support and facilitate flexible work arrangements as we shift to a hybrid way of working, this may include but is not limited to policies regarding the proportion of time in the office versus working from home, flexible work hours, and guidelines regarding the organisation of meetings, for example, all meetings are held as hybrid events to enable all to participate regardless of work location; regular, timely, and improved communications to foster a sense of mattering, connectedness and belonging; availability of and access to human resources supports including providing resources to facilitate improved leadership; constructive supervisor behaviours which enable open communication; and access to instrumental and emotional supports from colleagues and employers through formal and informal opportunities. These implications for practice are similar to those reported prior to the pandemic [22, 47] suggesting, despite the vastly different contexts under which WFH occurred, employers need to adopt strategies to meet the needs of employees irrespective of work location, as hybrid ways of working become more common place and we shift away from the notion that WFH is the exception, negotiated on a case by case basis.

The longitudinal design of this study is a key strength, with three waves of data over a 12-month time frame during the COVID 19 pandemic when Victorians who could WFH were required to do so. The study design enabled investigation of how sense of community and social support in the workplace changed over time and the impact on self-rated general health. Self-rated general health, sense of community, and social support were measured using validated instruments, adding to the strengths of this study. However, data were not available on participants’ sense of community, social support, or self-rated general health prior to the COVID 19 pandemic, thus it is unclear if observed levels of sense of community, social support, and self-rated health are congruent with pre-pandemic levels among employees who WFH. Further, it is not known what strategies organisations adopted to support employees’ sense of community or social support. Qualitative research is needed to explore what types of strategies were implemented by organisations. The convenience sample, the higher proportion of females compared with males in the sample (consistent with other COVID-19 research), and retention rate may limit the generalisability of the findings and as such the results should be interpreted with caution. There is no population data currently available for Victoria, Australia to compare our sample regarding the characterises of those working from home. Data from the Australian Bureau of Statistics (ABS) for August 2021 indicates approximately 41% of employed people worked from home regularly, of which two-thirds were managers or professionals who usually worked from home [48]. Similarly, 59% and 17% of our sample were professionals or managers respectively and as such our sample is likely reflective of professionals and managers in Australia, noting the ABS data includes all states and territories of Australia, and not just Victoria so it is possible given the varying public health restrictions across the country, our sample over or underestimates those working from home. A final limitation of this study is that employees are not randomly assigned to tasks or jobs, so job or task differences related to sense of community that may influence self-rated general health (e.g., high involvement management [12]) may not have been captured. Such practices and concepts should be considered in future research.

### Conclusion

Sense of community and social support are important determinants of employees’ general health and as such strategies to improve sense of community and social support are required in the workplace regardless of the where the work is done, from home, in the office, or both. This study provides important insights into considerations required by organisation to support WFH as we shift to more flexible hybrid models of work that incorporate options to WFH as standard practice. Organisations will need to facilitate opportunities to develop communities and provide appropriate support when employees are not co-located with their colleagues and managers. The current research contributes to an emerging understanding of the importance of workplace sense of community and social support and their association with self-rated general health. Further research is required to understand the strategies adopted by organisations to address sense of community and social support in the workplace, including factors associated with high involvement management.

## Supplementary Information

**Additional file 1: Figure S1.** Trajectories of work location over the three waves. **Table S1.** Model fit indices for GMM to determine trajectories of sense of community and social support.

## Data Availability

The datasets used and/or analysed during the current study are available from the corresponding author on reasonable request.

## References

[1] World Health Organization. WHO Director-General’s opening remarks at the media briefing on COVID-19–11 March 2020. 2020. Available from: https://www.who.int/director-general/speeches/detail/who-director-general-s-opening-remarks-at-the-media-briefing-on-covid-19---11-march-2020. Accessed June 2022.

[2] Di Martino V, Wirth L, Telework (1990). Telework. A new way of working and living. Int’l Lab Rev.

[3] Carillo K, Cachat-Rosset G, Marsan J, Saba T, Klarsfeld A (2021). Adjusting to epidemic-induced telework: empirical insights from teleworkers in France. Eur J Inform Syst.

[4] Victorian State Government (2021). Victoria’s Roadmap: delivering the National Plan.

[5] Charalampous M, Grant CA, Tramontano C, Michailidis E (2019). Systematically reviewing remote e-workers’ well-being at work: a multidimensional approach. Eur J Work Organ Psychol.

[6] Aguilera A, Lethiais V, Rallet A, Proulhac L (2016). Home-based telework in France: characteristics, barriers and perspectives. Transp Res Part A: Policy Pract.

[7] Klein KJ, D’Aunno TA (1986). Psychological sense of community in the workplace. J Community Psychol.

[8] McMillan DW (1996). Sense of community. J Community Psychol.

[9] McMillan DW. Sense of community, a theory not a value: a response to Nowell and Boyd J Community Psychol. 2011;39:507–19.

[10] McMillan DW, Chavis DM (1986). Sense of community: a definition and theory. J Commun Psychol.

[11] Garrett LE, Spreitzer GM, Bacevice PA (2017). Co-constructing a sense of community at work: the emergence of community in coworking spaces. Organ Stud.

[12] Böckerman P, Bryson A, Ilmakunnas P (2012). Does high involvement management improve worker wellbeing?. J Econ Behav Organ.

[13] Burr H, Berthelsen H, Moncada S, Nübling M, Dupret E, Demiral Y (2019). The third version of the Copenhagen psychosocial questionnaire. Saf Health Work.

[14] Boyd NM, Nowell B (2017). Testing a theory of sense of community and community responsibility in organizations: an empirical assessment of predictive capacity on employee well-being and organizational citizenship. J Community Psychol.

[15] Boyd N, Nowell B, Yang Z, Hano MC (2018). Sense of community, sense of community responsibility, and public service motivation as predictors of employee well-being and engagement in public service organizations. Am Rev Public Adm.

[16] Asante JO, Li MJ, Liao J, Huang YX, Hao YT (2019). The relationship between psychosocial risk factors, burnout and quality of life among primary healthcare workers in rural Guangdong province: a cross-sectional study. BMC Health Serv Res.

[17] Morganson VJ, Major DA, Oborn KL, Verive JM, Heelan MP. Comparing telework locations and traditional work arrangements: differences in work-life balance support, job satisfaction, and inclusion. J Managerial Psychol. 2010;25(6):578–95.

[18] Evanoff BA, Strickland JR, Dale AM, Hayibor L, Page E, Duncan JG (2020). Work-related and personal factors associated with mental well-being during the COVID-19 response: survey of health care and other workers. J Med Internet Res.

[19] Kossek EE, Pichler S, Bodner T, Hammer LB (2011). Workplace social support and work-family conflict: a meta-analysis clarifying the influence of general and work-family-specific supervisor and organizational support. Pers Psychol.

[20] Bavik YL, Shaw JD, Wang X-H (2020). Social support: multidisciplinary review, synthesis, and future agenda. Acad Manag Ann.

[21] Reznik J, Hungerford C, Kornhaber R, Cleary M. Home-based work and ergonomics: physical and psychosocial considerations. Issues Ment Health Nurs. 2021;43(10):975–79. 10.1080/01612840.2021.1875276 33571037

[22] Oakman J, Kinsman N, Stuckey R, Graham M, Weale V (2020). A rapid review of mental and physical health effects of working at home: how do we optimise health?. BMC Public Health..

[23] Vander Elst T, Verhoogen R, Sercu M, Van den Broeck A, Baillien E, Godderis L (2017). Not extent of Telecommuting, but job characteristics as proximal predictors of work-related well-being. J Occup Environ Med..

[24] Bentley TA, Teo STT, McLeod L, Tan F, Bosua R, Gloet M (2016). The role of organisational support in teleworker wellbeing: a socio-technical systems approach. Appl Ergon.

[25] Foy T, Dwyer RJ, Nafarrete R, Hammoud MSS, Rockett P (2019). Managing job performance, social support and work-life conflict to reduce workplace stress. Int J Productivity Perform Manage.

[26] Bernal D, Campos-Serna J, Tobias A, Vargas-Prada S, Benavides FG, Serra C (2015). Work-related psychosocial risk factors and musculoskeletal disorders in hospital nurses and nursing aides: a systematic review and meta-analysis. Int J Nurs Stud.

[27] Labrague LJ, De los Santos JAA (2020). COVID-19 anxiety among front-line nurses: predictive role of organisational support, personal resilience and social support. J Nurs Adm Manag.

[28] Charoensukmongkol P, Phungsoonthorn T (2021). The effectiveness of supervisor support in lessening perceived uncertainties and emotional exhaustion of university employees during the COVID-19 crisis: the constraining role of organizational intransigence. J Gen Psychol.

[29] Savolainen I, Oksa R, Savela N, Celuch M, Oksanen A (2021). COVID-19 Anxiety—A longitudinal survey study of psychological and situational risks among finnish workers. Int J Environ Res Public Health.

[30] Wang B, Liu Y, Qian J, Parker SK (2021). Achieving effective remote working during the COVID-19 pandemic: a work design perspective. Appl Psychol.

[31] Jylhä M (2009). What is self-rated health and why does it predict mortality? Towards a unified conceptual model. Soc Sci Med.

[32] Idler EL, Benyamini Y (1997). Self-rated health and mortality: a review of twenty-seven community studies. J Health Soc Behav.

[33] Wu S, Wang R, Zhao Y, Ma X, Wu M, Yan X (2013). The relationship between self-rated health and objective health status: a population-based study. BMC Public Health.

[34] Jylhä M, Volpato S, Guralnik JM (2006). Self-rated health showed a graded association with frequently used biomarkers in a large population sample. J Clin Epidemiol.

[35] Australian Institiute of Health and Welfare (2022). Social determinants of health.

[36] Wilkinson RG, Marmot M, World Health Organization (1998). Regional Office for Europe. The solid facts: social determinants of health.

[37] Oakman J, Kinsman N, Lambert K, Stuckey R, Graham M, Weale V (2022). Working from home in Australia during the COVID-19 pandemic: cross-sectional results from the employees working from home (EWFH) study. BMJ Open.

[38] Australian Bureau of Statistics. ANZSCO - Australian and New Zealand Standard Classification of Occupations. 2022. Available from: https://www.abs.gov.au/statistics/classifications/anzsco-australian-and-newzealand-standard-classification-occupations/latest-release. Accessed Nov 2022.

[39] Oakman J, Neupane S, Kyrönlahti S, Nygård C-H, Lambert K (2022). Musculoskeletal pain trajectories of employees working from home during the COVID-19 pandemic. Int Arch Occup Environ Health.

[40] Proust-Lima C, Philipps V, Liquet B. Estimation of extended mixed models using latent classes and latent processes: the R Package lcmm. J Stat Software. 2017;78(2):1–56.

[41] Christensen RHB. Ordinal - Regression models for ordinal data. R package version 2019. 12-10. ed2019. Available from: https://CRAN.R-project.org/package=ordinal.

[42] Syrek C, Kühnel J, Vahle-Hinz T, de Bloom J (2022). Being an accountant, cook, entertainer and teacher—all at the same time: changes in employees’ work and work-related well-being during the coronavirus (COVID-19) pandemic. Int J Psychol.

[43] Moens E, Lippens L, Sterkens P, Weytjens J, Baert S (2022). The COVID-19 crisis and telework: a research survey on experiences, expectations and hopes. Eur J Health Econ.

[44] Oakman J, Kinsman N, Graham M, Stuckey R, Weale V (2022). Strategies to manage working from home during the pandemic: the employee experience. Ind Health..

[45] Bolisani E, Scarso E, Ipsen C, Kirchner K, Hansen JP (2020). Working from home during COVID-19 pandemic: lessons learned and issues. Manage Mark Challenges Knowl Soc.

[46] Collins AM, Hislop D, Cartwright S (2016). Social support in the workplace between teleworkers, office-based colleagues and supervisors. New Technol Work Employ.

[47] Bjärntoft S, Hallman DM, Zetterberg C, Larsson J, Edvinsson J, Jahncke H (2021). A participatory approach to identify key areas for sustainable work environment and health in employees with flexible work arrangements. Sustainabil.

[48] Australian Bureau of Statistics. Working arrangements, ABS. 2021. Available from: https://www.abs.gov.au/statistics/labour/earnings-and-working-conditions/working-arrangements/latest-release. Accessed Nov 2022.

