# Supplementary material for: Working from home during the COVID 19 pandemic: a longitudinal examination of employees’ sense of community and social support and impacts on self-rated health
Source: BMC Public Health. 2023 Jan 3;23:11. doi: 10.1186/s12889-022-14904-0 (PMC9808765; doi:10.1186/s12889-022-14904-0)
Supplement: Supplementary file 1 — Additional file 1: Figure S1. Trajectories of work location over the three waves. Table S1. Model fit indices for GMM to determine trajectories of sense of community and social support. [file 12889_2022_14904_MOESM1_ESM.docx]

# **Additional Materials**


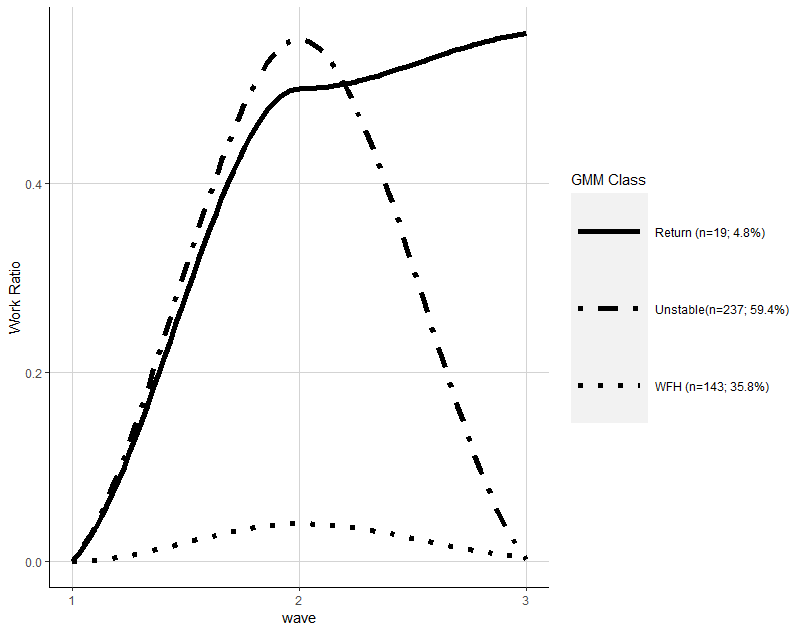


Figure S1: Trajectories of work location over the three waves

Table S1: Model fit indices for GMM to determine trajectories of sense of community and social support

|  | Groups | loglik | BIC | SABIC | entropy | %class1 | %class2 | %class3 | %class4 | %class5 |
| --- | --- | --- | --- | --- | --- | --- | --- | --- | --- | --- |
| Sense of community | 1 | -1554.13 | 3134.283 | 3121.583 | 1 | 100 |  |  |  |  |
|  | 2 | -1515.64 | 3076.827 | 3054.602 | 0.761651 | 15.80402 | 84.19598 |  |  |  |
|  | 3 | -1515.64 | 3096.344 | 3064.594 | 0.321714 | 0 | 83.8565 | 16.1435 |  |  |
|  | 4 | -1515.64 | 3115.862 | 3074.586 | 0.220983 | 0 | 18.0867 | 81.9133 | 0 |  |
|  | 5 | -1515.64 | 3135.379 | 3084.578 | 0.176968 | 0 | 0 | 19.58146 | 80.41854 | 0 |
| Social support | 1 | -1556.79 | 3139.563 | 3126.863 | 1 | 100 |  |  |  |  |
|  | 2 | -1503.17 | 3051.799 | 3029.574 | 0.752513 | 22.3565 | 77.6435 |  |  |  |
|  | 3 | -1478.51 | 3021.979 | 2990.229 | 0.722441 | 13.8889 | 73.6111 | 12.5000 |  |  |
|  | 4 | -1478.51 | 3041.465 | 3000.19 | 0.470927 | 72.80967 | 16.46526 | 10.72508 | 0 |  |
|  | 5 | -1478.51 | 3060.951 | 3010.15 | 0.341928 | 16.76737 | 0 | 11.02719 | 72.20544 | 0 |
